# High frequency monitoring for impact assessment of temperature, oxygen and radiation in floating photovoltaic system

**DOI:** 10.1038/s41598-025-96257-3

**Published:** 2025-06-05

**Authors:** Matheus Kopp Prandini, Rafael de Carvalho Bueno, Jucimara Andreza Rigotti, Tobias Bleninger, Michael Mannich, Luis Henrique Novak

**Affiliations:** 1 Department of Environmental Engineering-DEA, Federal University of Paraná-UFPR, Curitiba, PR 81531-990 Brazil; 2 Postgraduate Program in Water Resources and Environmental Engineering, Federal University of Paraná-UFPR, Curitiba, PR 81531-990 Brazil; 3 Paraná State Water and Sanitation Company-SANEPAR, Rua Engenheiros Rebouças, 1376, Rebouças, 80215-900 Brazil

**Keywords:** Limnology, Environmental impact, Renewable energy

## Abstract

Floating photovoltaic (FPV) systems are designed for free water surface installations to provide a feasible solution for places with no availability of land areas and to avoid land-use conflicts caused by conventional solar energy farms. However, lakes and reservoirs are essential for ecosystem services like water supply and biodiversity support. In this regard, there was a lack of long-term and high-frequency monitoring data of important parameters influenced by FPV installation such as photosynthetically active radiation (PAR) and dissolved oxygen (DO). Temperature, despite being more reported, there is no consensus on increase or decrease values below the FPV. In this study, we conduct a comprehensive field assessment to accurately quantify temperature variations (at the weather station, modules, between the modules and the water surface and within the water), DO, and PAR of a FPV installed in a water supply reservoir. High-frequency monitoring sensors indicated that despite the 94.7% of radiation reduction below the FPV compared to the lake reference station, slight differences in water temperature and dissolved oxygen were found between measurements in monthly and daily averages in a system that covers less than 1% of the reservoir water surface. In addition, a microclimate due to the module warming is created between the module and water surface with temperature 12% greater than the weather station measurements. The fluctuation throughout the different time frequencies showed that the processes of the reservoirs are influenced by the FPV according to complex interactions among meteorological conditions, FPV configuration, and site-specific factors.

## Introduction

Solar energy is widely recognized as a promising renewable source because of its abundance and sustainability. However, it still has certain environmental impacts^1–3^. Traditional photovoltaic systems typically require large land areas for installation, often resulting in significant ecological impacts, such as vegetation removal and land use conflicts related to territorial and landscape concerns^4^. As an alternative, Floating Photovoltaic (FPV) systems, designed for deployment on water surfaces, offer a viable solution for lakes, ponds, reservoirs, dams, and pits. By reducing land use requirements, FPV systems help mitigate some of these environmental concerns^1,4,5^.

Most FPV studies focus on energy generation and construction-related aspects^6,7^. However, lakes and reservoirs are vital aquatic ecosystems that provide drinking water, hydroelectric power, and support rich biodiversity^8^. In addition, freshwater environments are considered important for climate change due to their sensitivity to environmental and climatic fluctuations^9^. Despite this, research on the effects of FPV on aquatic ecosystems, particularly its influence on changes in the mixed layer of the surface, remains limited and warrants further investigation^10–12^.

Research on the effect of FPV on water temperature in aquatic environments focuses mainly on the efficiency of the system^13,14^. For example, countries such as Brazil and Canada could meet their 2050 solar energy targets by covering less than 10% of reservoir surfaces with floating solar panels^4^. Although studies have explored the feasibility of FPV^15–19^, they have yet to comprehensively assess its environmental impacts through an integrated approach^20^.

Previous studies have highlighted the need for further research on potential temperature changes^1,21^. A comprehensive review suggested that FPV can reduce water temperature by attenuating solar radiation in the water column through a shading effect^1^. A pilot study conducted in a stormwater pond in the Netherlands observed a temperature decrease of approximately 0.2 $^{\circ }$C at the FPV site during spring, 0.8 $^{\circ }$C in summer and negligible differences in winter (< 0.1 $^{\circ }$C)^22^. Similarly, FPV-covered lakes exhibited significantly lower daily average temperatures and reduced water temperature fluctuations^23^. In contrast, a field study in shallow water reservoirs in Singapore reported a temperature increase of 0.47 $^{\circ }$C during the day and 0.51 $^{\circ }$C at night beneath a 1 hectare FPV system^24^. Meanwhile, no significant temperature differences were found between three pilot FPV systems and the control site in a brackish lake in Oostvoorne, Netherlands^25^.

The results of FPV modeling in a fish tank indicated that, with approximately 40% surface coverage, the water surface below FPV decreased by an average of 0.30 $^{\circ }$C over the day and night periods in summer and winter^26^. The authors also reported seasonal reductions in mean values, with a temperature drop of 0.77 $^{\circ }$C in winter and 1.4 $^{\circ }$C in summer. Daily variations ranged from 0.25 $^{\circ }$C at night to 0.28 $^{\circ }$C during the day in winter, and from 0.30 $^{\circ }$C at night to 0.38 $^{\circ }$C during the day in summer. These findings are consistent with observed temperature decreases of up to 2.8 $^{\circ }$C in FPV systems^27^. In addition, no temperature differences were identified at a depth of 10 $\textrm{m}$, with a slight tendency to a higher temperature at the FPV site^27^.

FPVs can affect dissolved oxygen concentrations by acting as barriers to wind and solar radiation, which reduces reaeration and limits dissolved oxygen production by photosynthetically active organisms^19,28^. The literature emphasizes eutrophication reduction, particularly the decline of toxic microalgae, as a key in evaluating FPV impacts^29,30^. The shading effect of FPV can decrease algae growth, as aquatic organisms rely on available radiant energy within the water column. Reduced shortwave radiation at the water surface diminishes photosynthetically active radiation (PAR), consequently lowering primary production rates^10^. Photosynthetically active radiation, which includes wavelengths from 400 $\textrm{nm}$ to 700 $\textrm{nm}$^7,31^, is essential for phytoplankton. Variations in PAR may force phytoplankton species to adapt, affecting both biomass and species richness^11^.

There is currently limited consensus on the impacts of FPV on aquatic environments. While some benefits have been identified, such as reduced evaporation, moderated water temperature, control of toxic algae, and enhanced efficiency of photovoltaic modules, these advantages may not fully capture the complexity of FPV effects. Although these factors underscore the potential for mitigation in specific contexts, a comprehensive understanding of the medium- and long-term environmental impacts remains lacking. The balance between the positive and negative environmental effects of FPV systems presents a substantial challenge for scientific research, technological advancement, and global deployment^4^. High-frequency monitoring studies are essential to examine variations in temperature profiles, photosynthetically active radiation, and daily fluctuations in these parameters, offering critical insights into FPV impacts on lake limnology and ecosystem dynamics. Given the scarcity of studies using such high-resolution data in the long term, this study aims to conduct comprehensive field assessments to accurately quantify these variations. We hypothesize that FPV impacts photosynthetically active radiation through a shading effect, which may subsequently lead to variations in temperature and dissolved oxygen concentrations. We further hypothesize that these changes are not binary (increase/decrease), but rather fluctuate throughout the day, influenced by complex interactions among meteorological conditions, FPV configuration, and site-specific factors.

## Methods

### Study site and FPV design

**Fig. 1:**
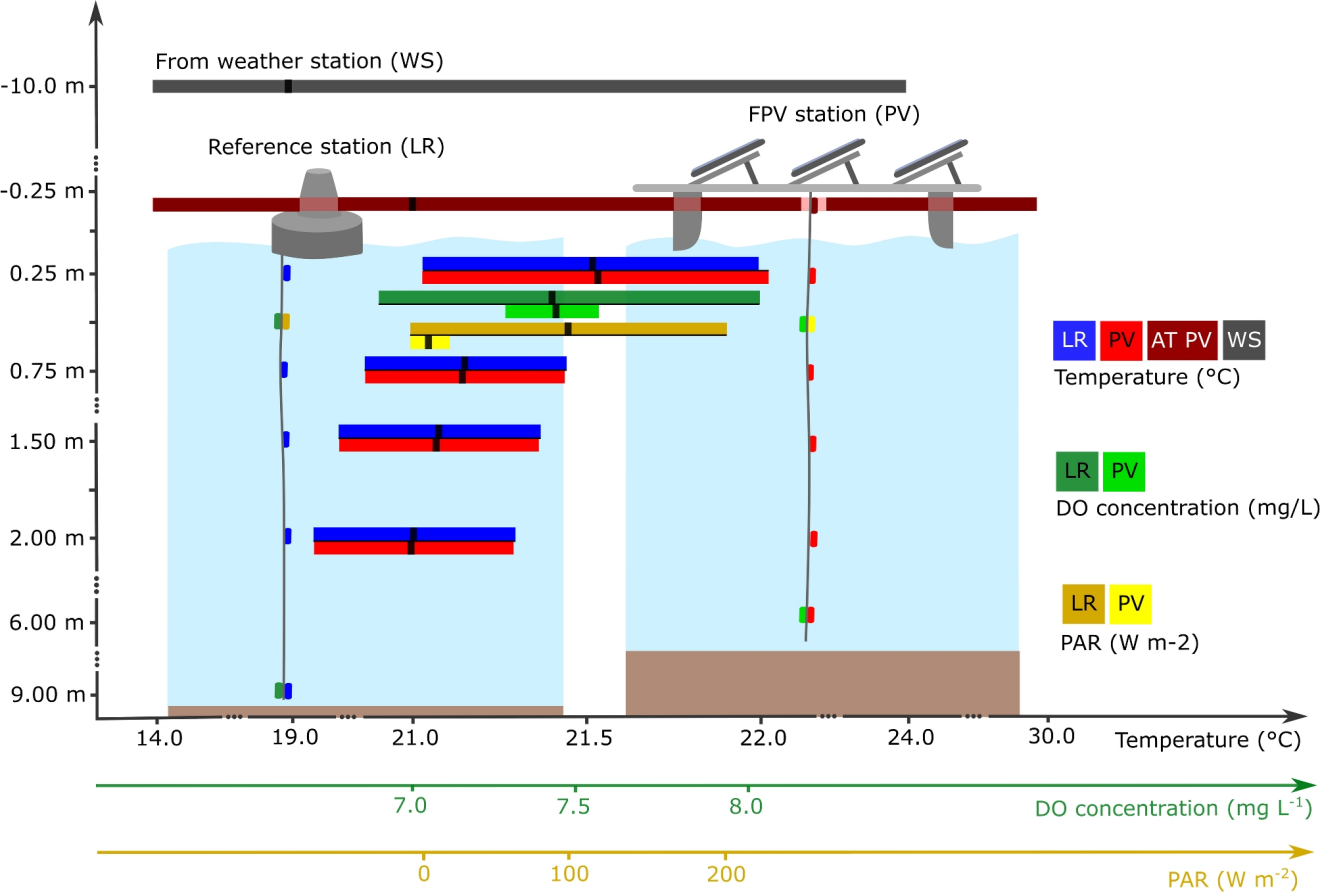
Overview of monitoring system. In which: LR is the reference station, PV is the FPV station, AT PV is the temperature sensor below the photovoltaic modules between the air-water interface and the panels, WS is the temperature sensor at the weather station, DO is Dissolved Oxygen (green), and PAR is the sensor for Photosynthetically Active Radiation (yellow). Bars indicate the 95% confidence interval. Black dots in the center of the bars indicate the average values.

**Fig. 2:**
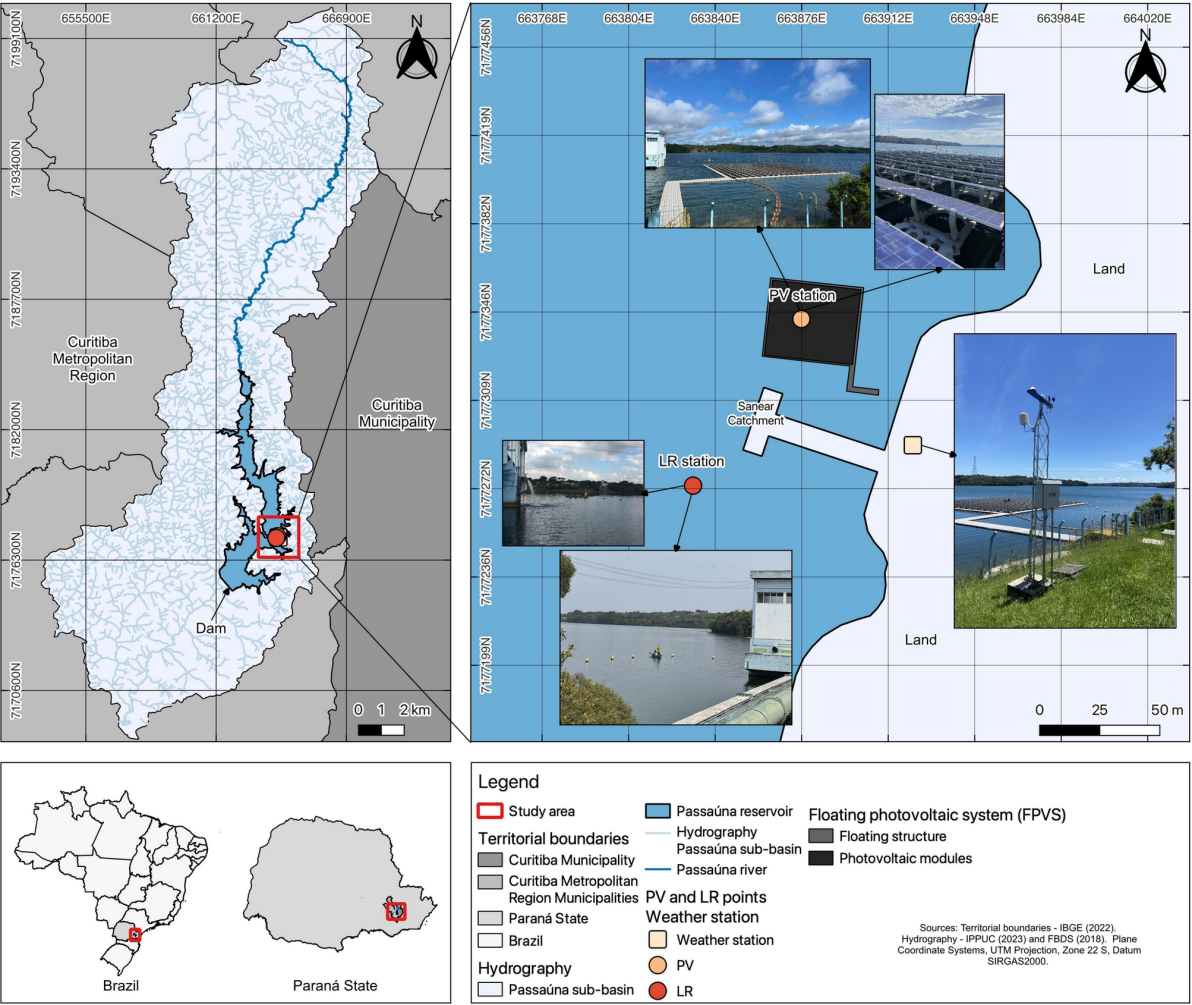
Study area at Passaúna reservoir. Localization of the measurement stations PV (middle of FPV system), LR (lake reference point), and the weather station. The map was created with QGIS^35^ version 3.28.14. Source: geospatial data on hydrography and watershed from IPPUC^36^ and FBDS^37^, and municipalities from IBGE^38^.

A floating photovoltaic pilot system was monitored from October 2022 to February 2024 (Fig. 1). The FPV covers about 0.02% of the total surface area of the reservoir, which is an FPV area of 1326 $\textrm{m}^{2}$. Passaúna Reservoir (Fig. 2) is a drinking water supply reservoir located in the southern region of Brazil near Curitiba (${25}^{\circ }$31’ S, ${49}^{\circ }$23’ W). The reservoir has a surface area of about 8.7 $\textrm{km}^2$ and a maximum water depth of 17 $\textrm{m}$. The climate is characterized by temperate conditions with no dry season and a warm summer, it is classified as Cfb according to the Köppen-Geiger system^32^. The mean temperature varies from 5.8 $^{\circ }$C to 33.8 $^{\circ }$C. Annual precipitation in the region ranges between 1400 $\textrm{mm}$ and 1600 $\textrm{mm}$^33^. The area is dominated by diurnal south-easterly winds, with an average of 1.52 $\textrm{ms}^{-1}$. Wind events exceeding 2.00 $\textrm{ms}^{-1}$ are rare and typically occur due to isolated weather events.

The pilot system has a maximum energy capacity of 130 kWp^34^, similar to most floating photovoltaic pilot power plants installed worldwide^19^. The system consists of 396 polycrystalline silicon photovoltaic panels of 330 W. It is arranged in 22 strings, each containing 18 photovoltaic panels. The photovoltaic panels are installed on a floating structure and connected to the shore via a floating walkway. The floating structure is composed of 1009 floats of type 1 (including the access walkway) and 396 floats of type 2 (Supplementary information).

### Measurements and data

To assess the impact of the FPV on water temperature, dissolved oxygen concentration, and availability of photosynthetically active radiation, the study site was monitored at two distinct monitoring stations through continuous measurements. The first continuous monitoring station (PV) was positioned at the center of the FPV (Fig. 2). To monitor the temperature profile, a vertical thermistor chain of six temperature loggers (Minilog II-T, Vemco, precision 0.01 $^{\circ }$C, accuracy ± 0.1 $^{\circ }$C) was installed. The thermistors were deployed at depths of 0.25 $\textrm{m}$, 0.75 $\textrm{m}$, 1.50 $\textrm{m}$, and 2.00 $\textrm{m}$, with a sampling interval of one minute. Additionally, a sensor was installed between the water surface and the panel at the same PV location, with a Minilog II-T (Vemco) at a sampling interval of 1 m. Furthermore, the module temperature below the panel was measured by a SFCS-50 (Pt-100, Class B) sensor (detailed localization of the six sensors are in the supplementary information). A continuous monitoring station (LR) was strategically positioned in open water (Fig. 1). The monitoring stations were selected through evidence of previous literature modeling results and previous field measurements. A pilot field campaign was carried out at many profiles below the FPV system, as well as at different distances from the system to investigate the potential influence of the FPV on water quality characteristics with respect to spatial variations. The location of the LR station was selected to ensure it was far enough to avoid influence from the FPV, yet close enough to represent the conditions of the monitored area accurately. A thermistor chain of six temperature loggers was also installed in the LR station at the same depths as at the PV station.

Dissolved oxygen and temperature sensors (miniDOT, precision 0.01 $\textrm{mg}\, L^{-1}$, accuracy ± 0.3 $\textrm{mg}\, L^{-1}$; precision 0.01 $^{\circ }$C, accuracy ± 0.1 $^{\circ }$C) were installed at a depth of 0.50 $\textrm{m}$ at both PV and LR monitoring stations, as well as at 6.00 $\textrm{m}$ at PV and at 9.00 $\textrm{m}$ at LR station. The miniDOTs were equipped with an anti-fouling kit to reduce biofilm growth (Precision Measurement Engineering, Inc.). Photosynthetically active radiation sensors (miniPAR, precision 1 $\upmu \mathrm{mol\, s}^{-1} \textrm{m}^{-2}$, accuracy 4 ${\upmu {A}}$ per 1000 $\upmu \mathrm{mol\, s}^{-1} \textrm{m}^{-2}$ were installed at the same locations where the miniDOT sensors were deployed, but only at the water surface (0.50 $\textrm{m}$) at both monitoring stations. The photosynthetically active radiation was measured in the central part of the FPV. Additionally, relative humidity (MeteoTemp, precision 0.1 %, accuracy ± 1.8 %), air temperature (MeteoTemp, precision 0.1 $^{\circ }$C, accuracy ± 0.2 $^{\circ }$C), downwelling shortwave radiation (MS-80, precision 1 $\textrm{W} m^{-2}$, accuracy ± 1 $\textrm{W} m^{-2}$), wind direction (MeteoWind, precision 1$^\circ$, accuracy 2$^\circ$), and wind speed (MeteoWind, precision 0.01$\textrm{ms}^{-1}$, accuracy ± 0.05 $\textrm{ms}^{-1}$) were measured at 10 $\textrm{m}$ by a weather station based on shore and located 40 $\textrm{m}$ away from the FPV, with data recorded at 1 min intervals.

### Data processing and analysis

Data analysis procedures were performed for eight continuously monitored parameters: air temperatures, wind speed, air-water interface temperature, module temperature, water temperature, dissolved oxygen, solar radiation, and photosynthetically active radiation. These parameters were measured by the weather station or by sensors at PV and LR stations. To ensure data quality, all time series were quality-checked, which included the removal of spurious data, such as measurements taken during maintenance periods and data identified as anomalies. Anomalies were identified using a specialized filter to detect abrupt differences between two measurements within a predefined time window. For the water temperature, the time window was set to 15 min, while for dissolved oxygen, it was set to 30 min, reflecting the natural temporal variability of each parameter. Furthermore, as the manuscript focuses on the effect of the FPV system based on measurements performed at the PV and LR stations, any period of data identified as an outlier in either of the monitored stations was excluded from the analysis at both stations. The subsequent data sets were then averaged over 5 min intervals.

To compare how isotherms deepening and thermal stability differ from LR and PV stations, we used the Interwave Analyzer^39^ to calculate the hourly averaged buoyancy frequency. The buoyancy frequency, derived from instrumented stations, was used to investigate the vertical density gradients at both LR and PV stations. The buoyancy frequency indicates the rate of change in potential density with depth, reflecting the stability of the water column in terms of potential density. We opted not to use Schmidt Stability, which measures the work required to mix a lake to an isothermal condition, as it could introduce bias when comparing stations with different depths. In addition, we did not compute the surface boundary layer deepening by analyzing the thermocline depth since Passaúna reservoir exhibits a continuous stratification profile characterized by a measly linear temperature decrease from the water surface to the bottom^40^. Therefore, we used the Interwave Analyzer^39^ to calculate the buoyancy frequency according to the equation:

1 $$\begin{aligned} {N}^{2} = -\frac{g}{\rho }\frac{\partial {\rho }}{\partial {z}} \end{aligned}$$

where *g* ($\textrm{m s}^{-1}$) is the gravitational acceleration, $\rho$ is the water density (kg m$^{-3}$)), and *z* is the depth measured from the surface ($\textrm{m}$).

To isolate spurious variations in temperature and dissolved oxygen and obtain more accurate estimates to analyze differences between LR and PV stations driven by solar radiation and wind forcing frequencies, we computed the power spectral densities (PSD) of underwater temperature at a depth of 0.25 $\textrm{m}$, dissolved oxygen concentration at 0.50 $\textrm{m}$, and wind speed estimated from the weather station. The PSDs were calculated using the Welch method, employing a Hamming window with a size of 30 d for temperature and 10 d for dissolved oxygen and wind speed. The calculations were performed using the software Interwave Analyzer^39^.

## Results

### Time series description

**Fig. 3:**
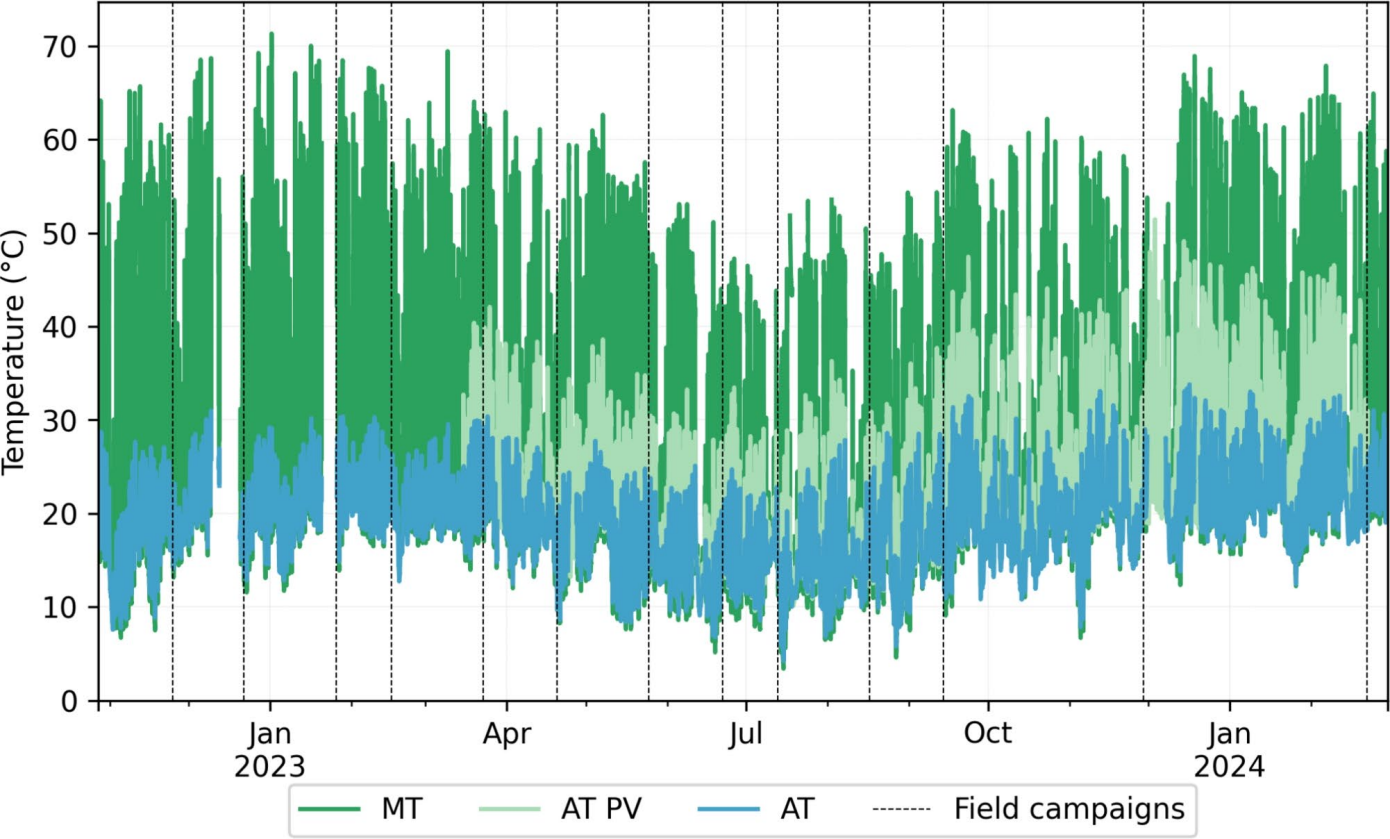
Time series of air temperatures measured at the weather station (AT), below the photovoltaic modules between the air-water interface and the panels (AT PV), and the module temperature (MT) from October 2022 to February 2024.

The time-series data of module temperature, air temperature measured at the weather station, and air temperature beneath the photovoltaic modules illustrates the thermal behavior of the environment surrounding the modules and the water surface (Fig. 3). The air temperature beneath the photovoltaic modules exhibits a higher average and standard deviation (21.4 $^{\circ }$C and 7.1 $^{\circ }$C) compared to the data from the weather station (18.9 $^{\circ }$C and 4.8 $^{\circ }$C), indicating that the air beneath the PV modules is significantly warmer. A more comprehensive overview of the environmental data obtained from the meteorological station can be found in the information.

**Fig. 4:**
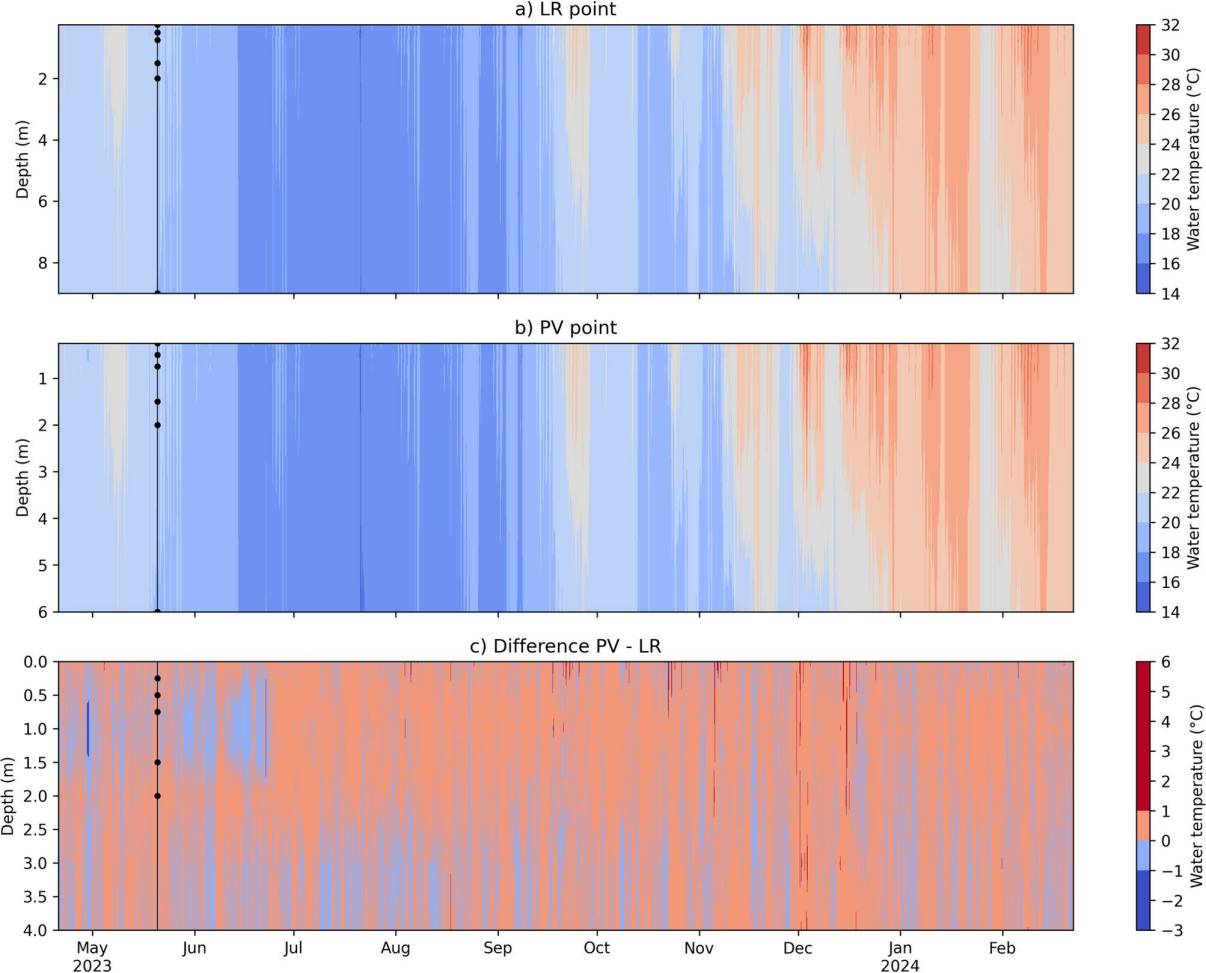
Temperature profiles and differences between monitoring stations. The water temperature profile is interpolated between measurement sensors at stations (**a**) LR and (**b**) PV. Panel (**c**) shows the differences in temperature values between stations. Positive values (red) indicate that the water temperature at station PV is higher than at station LR, while negative values (blue) indicate the opposite. Black dots show the position of the thermistors.

The water temperature profiles presented similar trends at both monitoring stations (Fig. 4). Despite the presence of the FPV system at the PV monitoring station, which may partially obstruct solar radiation and affect wind shear stress at the water surface, no significant differences were observed between PV and LR. The slight difference between PV and LR was more frequent between -1 $^{\circ }$C and 0 $^{\circ }$C (58.33 %) than 0 $^{\circ }$C and 1 $^{\circ }$C (38.25 %) (Fig. 4c). Moreover, differences greater than ± 1 $^{\circ }$C were observed only during 0.17% of the analyzed period.

**Fig. 5:**
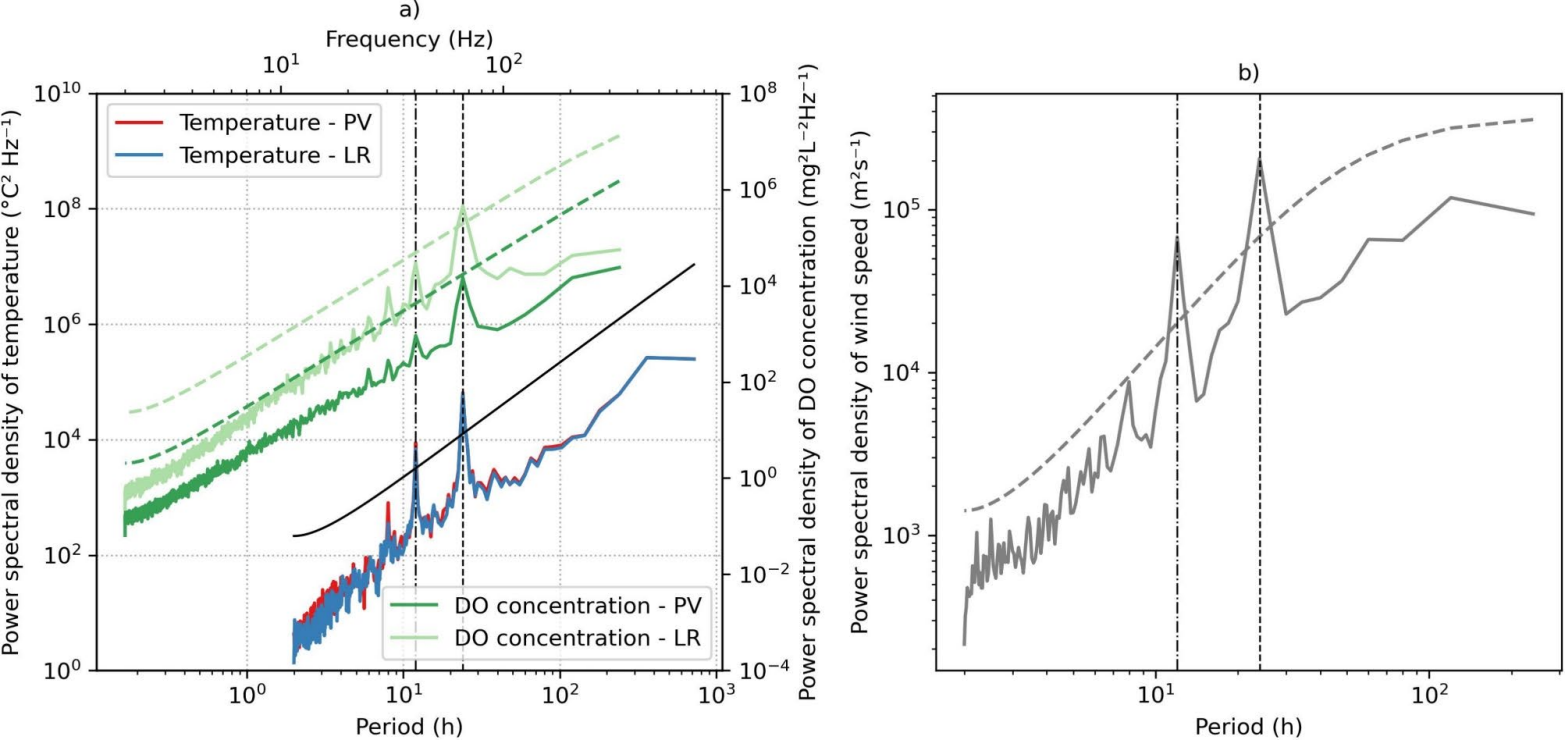
(**a**) Power spectral density of water temperature series at a 0.25 $\textrm{m}$ depth and dissolved oxygen concentration at 0.50 $\textrm{m}$ depth for stations PV and LR. (**b**) Power spectral density of wind speed measured at weather station. The black solid line and dashed greenish and gray lines show the 95% confidence limit of the mean red noise spectrum for the time series of temperature, dissolved oxygen concentration, and wind speed respectively. The dot-dashed and dashed vertical lines highlight the diurnal (24 h) and semidiurnal (12 h) components, respectively.

As the water temperature is influenced by periodic meteorological forcing such as wind speed and solar radiation, we applied the power spectral density of water temperature to eliminate irrelevant fluctuations unrelated to the differences between the LR and PV stations, highlighting only the dominant components of temperature fluctuations. The power spectral density of water temperature measured at a depth of 0.25 $\textrm{m}$ revealed a slightly higher peak at the semidiurnal period (12 h) at the PV site, approximately 2616.64 $^\circ$C$^{2}$ Hz$^{-1}$ higher compared to the LR monitoring point (Fig. 5a). For the diurnal period (24 h), the slightly higher peak was 23.84 $^\circ$C$^{2}$ Hz$^{-1}$ greater at the LR point. Additionally, the lower peak at the semidiurnal period (12 h period) reflects similar findings as the diurnal period, likely attributed to the harmonic effect of the temperature data, rather than describing a semidiurnal oscillatory pattern of the temperature time series.

**Fig. 6:**
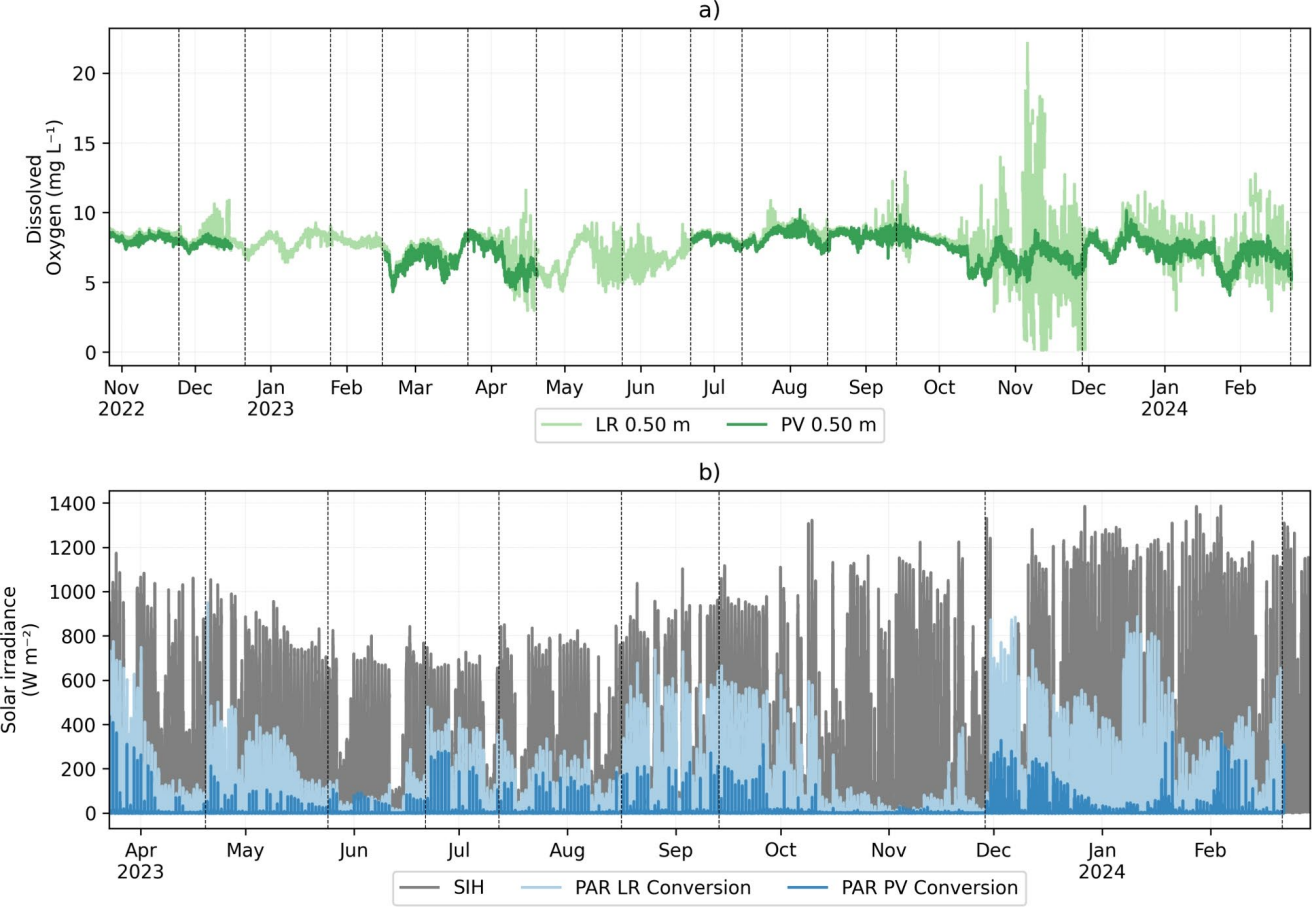
Time series of (**a**) dissolved oxygen and (**b**) solar irradiance horizontal (SIH) and photosynthetically active radiation (PAR) at stations PV and LR measured at 0.50 $\textrm{m}$ depth. PAR was converted from $\upmu$mol m$^{-2}$ s$^{-1}$ to W m$^{-2}$ a conversion factor of 2.1. Gray solid line is the solar radiation measured at the weather station. Vertical dashed-lines show periods of data collection and sensor maintenance campaigns.

Dissolved oxygen concentration exhibited a notable increase in variability, particularly during June, September, and November 2023 (Fig. 6a). The range from minimum to maximum values of DO for PV was 4.0 $\textrm{mg}\, L^{-1}$ to 10.2 $\textrm{mg}\, L^{-1}$ and for LR 2.9 $\textrm{mg}\, L^{-1}$ to 18.1 $\textrm{mg}\, L^{-1}$, leaving out the data variation assigned to the biofilm growth in the sensors in November 2023. Although the data suggest that the variability of dissolved oxygen at the LR station was higher than at the PV station, the mean value was not as significantly affected, showing a mean and standard deviation of 7.6 ± 0.9 $\textrm{mg}\, L^{-1}$ and 7.4 ± 1.7 $\textrm{mg}\, L^{-1}$ for PV and LR, respectively. This difference is below the accuracy range of the sensors (0.3 $\textrm{mg}\, L^{-1}$).

The solar radiation data from the weather station were used as a reference to illustrate the impact of reduced PAR caused by the shading effect of the FPV (Fig. 6b). The most significant changes were observed in photosynthetically active radiation (PAR), which had a significant reduction at the PV station compared to the LR station, with an average decrease of 94.7% relative to the measurements taken at the LR station.

### Time-frequency analysis

**Fig. 7:**
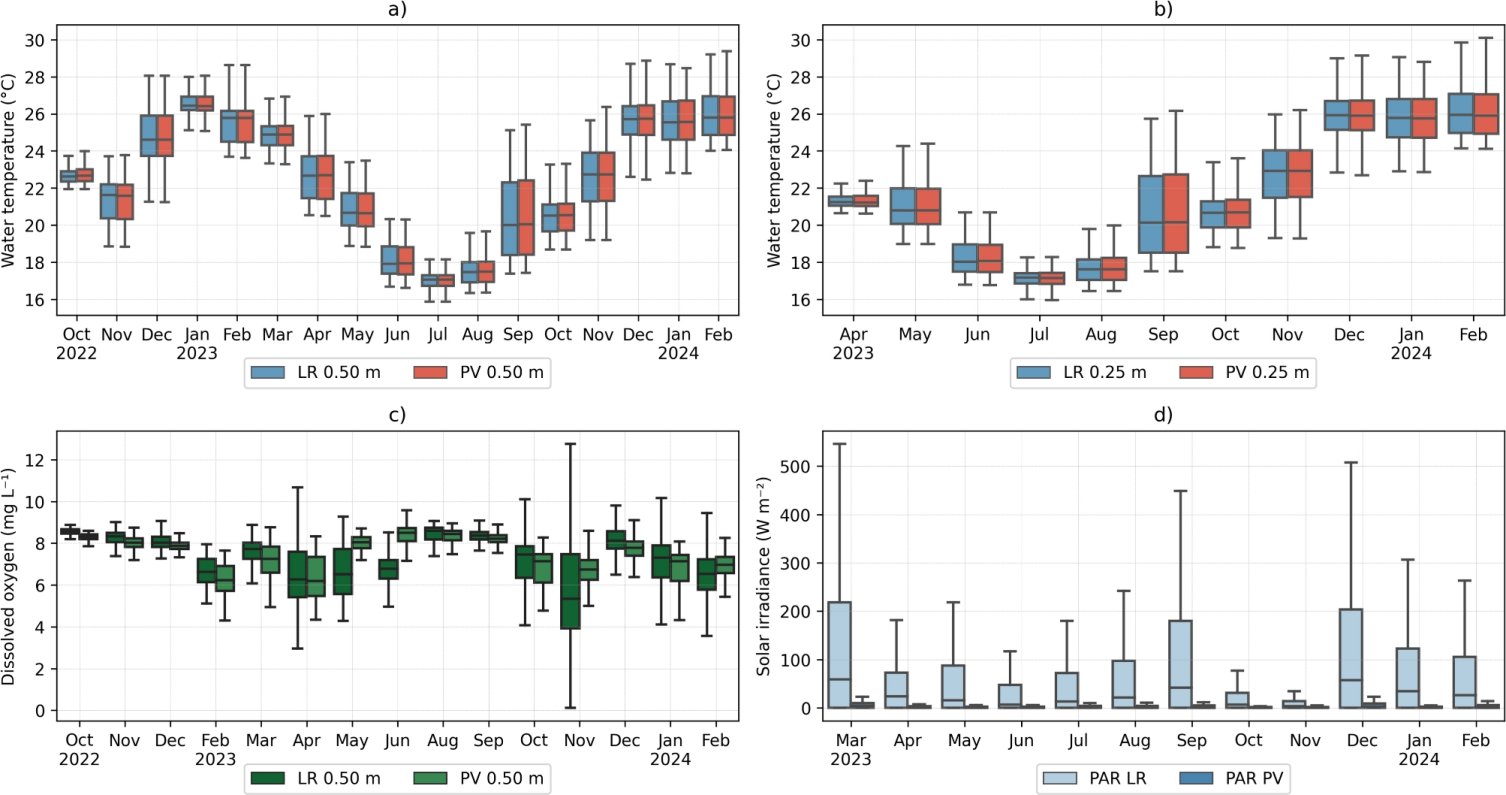
Comparison between monthly grouped water temperature, dissolved oxygen, and photosynthetically active radiation. Monthly boxplots (excluding outliers) of the water temperature at (**a**) 0.50 $\textrm{m}$ and (**b**) 0.25 $\textrm{m}$ depth, (**c**) dissolved Oxygen at 0.50 $\textrm{m}$ depth, and (**d**) photosynthetic active radiation at 0.50 $\textrm{m}$ depth on the PV and LR stations.

As changes in water temperature are primarily influenced by temporal variation (Fig. 5), we analyzed time-series data at different frequencies to better quantify the differences in water temperature, dissolved oxygen, and photosynthetically active radiation induced by the FPV system. Monthly averages of water temperatures across superficial depths consistently showed minimal differences between measurements taken at the PV and LR stations throughout the entire monitoring period, with a major difference in the mean values of 1.7 $^{\circ }$C of difference in January 2023 at 0.50 $\textrm{m}$ (Fig. 7a) and 1.5 $^{\circ }$C in April 2023 at 0.25 $\textrm{m}$ (Fig. 7b), with both showing higher temperatures at the PV station. Similarly, dissolved oxygen measured at 0.50 $\textrm{m}$ depth at the PV station indicated close values compared to measurements at the LR station (Fig. 7c). The months with more differences in the mean were May and June 2023, with 1.4 $\textrm{mg}\, L^{-1}$ and 1.2 $\textrm{mg}\, L^{-1}$, respectively. In both cases, the lower values were at the LR station compared to the DO concentration at the PV station. The most notable finding is the strong fluctuation of dissolved oxygen at the LR station. Although the highest variability was observed in November 2023, elevated fluctuations in dissolved oxygen were observed throughout the entire analyzed period. The standard deviation of dissolved oxygen was considerably higher in May 2023 at the LR station (1.2 $\textrm{mg}\, L^{-1}$) compared to the PV station (0.3 $\textrm{mg}\, L^{-1}$) and in February 2024 at the LR station (1.6 $\textrm{mg}\, L^{-1}$) compared to the PV station (0.5 $\textrm{mg}\, L^{-1}$).

The variability in temperature and dissolved oxygen concentration contrasts sharply with the significant decline in photosynthetically active radiation, which decreased meaningfully in the summer months, especially in March 2023 at the LR station (136.9 W m$^{-2}$) compared to the PV station (6.34 W m$^{-2}$) on average (Fig. 7d). The reduction is a result of the shading effect of the FPV, which blocked most parts of solar radiation at some points of the FPV system (Fig. 6b).

**Fig. 8:**
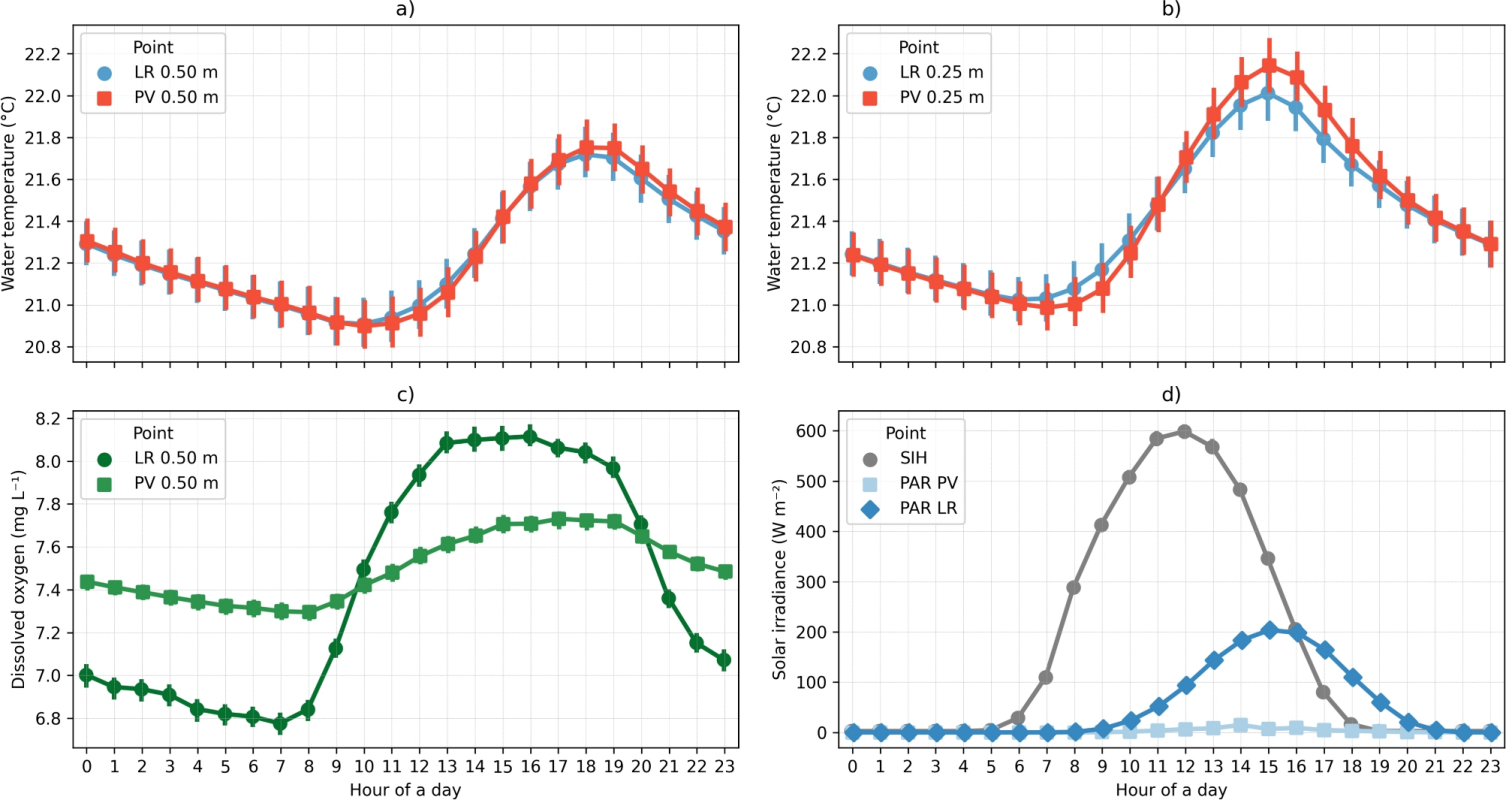
Comparison between daily grouped (**a**) water temperature at 0.50 $\textrm{m}$, (**b**) water temperature at 0.25 $\textrm{m}$, (**c**) dissolved oxygen, and (**d**) photosynthetically active radiation at 0.50 $\textrm{m}$ on Lake Reference-— LR and Floating Photovoltaic System — PV, and meteorological station data of Solar Irradiance Horizontal – SIH.

When the time series data were grouped into hourly averages over one day, the variation in water temperature between the two monitored stations became more apparent. Although the differences fall within the range of the sensor’s accuracy, the analysis reveals a faster warming of the water temperature at the LR station at 0.50 $\textrm{m}$ depth, although attenuated due to the increased depth of the water column (Fig. 8a) compared to the PV station. This effect was also observed at 0.25 $\textrm{m}$ during the morning (6 to 10 a.m.) (Fig. 8b). The highest values of temperature at 0.25 $\textrm{m}$ depth were 30.4 $^{\circ }$C and 30.3 $^{\circ }$C at stations PV and LR, respectively. The lowest temperature at 0.25 $\textrm{m}$ depth at both PV and LR stations was 16.0 $^{\circ }$C.

Throughout the day, the hourly average of dissolved oxygen exhibited pronounced fluctuations (Fig. 8c). The concentration was higher at the LR station only from 10 a.m. to 8 p.m., with a major difference of 0.4 $\textrm{mg}\, L^{-1}$. Additionally, the diurnal variability of dissolved oxygen under the influence of the FPV was attenuated, resulting in a daily average of 7.5 ± 0.9 $\textrm{mg}\, L^{-1}$ in the PV station, which contrasts with the high diurnal variability observed at the LR station of 7.4 ± 1.4 $\textrm{mg}\, L^{-1}$.

The photosynthetically active radiation is strongly affected by the FPV system (Fig. 8d). A particular interest is the time of day when radiation intensity peaks compared to the values recorded at the weather station. Additionally, at the LR station, radiation reaches its highest value of 203.8 W m$^{-2}$ at 3 p.m., whereas at the PV station, its peak of 14.9 W m$^{-2}$ occurs at 2 p.m.

### Mixing characterization

**Fig. 9:**
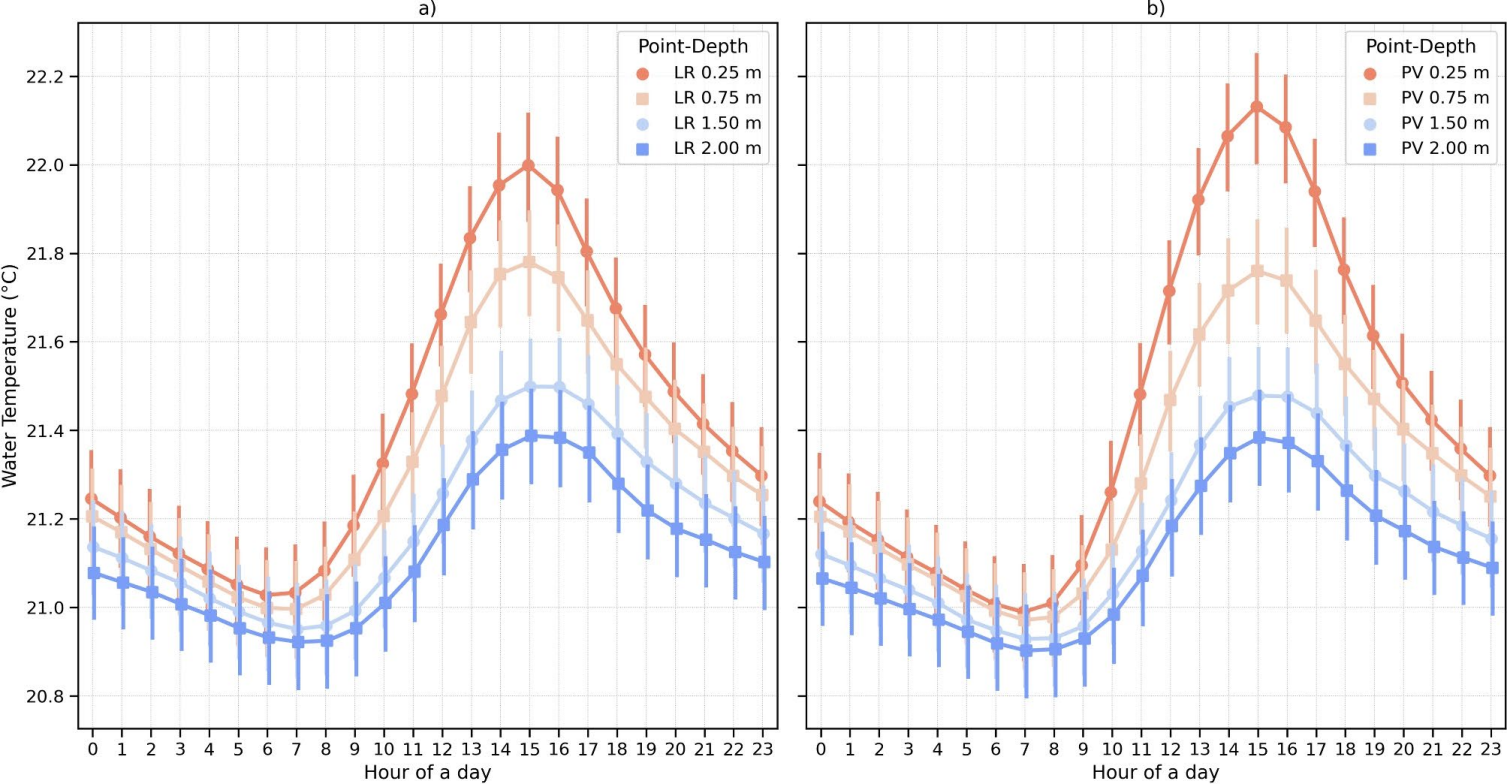
Hourly averages of temperature for LR (**a**) and PV (**b**) for the depths 0.25 $\textrm{m}$, 0.75 $\textrm{m}$, 1.50 $\textrm{m}$ and 2.00 $\textrm{m}$. The largest effect on the heating of water surface temperature is seen at the PV point at 0.25 $\textrm{m}$ (reduction of mixing in PV due to FPV). With increasing depth, LR shows higher temperatures (effect of shading in PV). Data are presented with a small temporal offset to avoid overlap of the data plot. A 0.1 offset was applied in the graphic to prevent overlap.

To characterize in detail the differences in mixing processes induced by the FPV, we analyzed the daily average water temperature over different depths for both stations (LR and PV). We observed that the surface water temperature at 0.25 $\textrm{m}$ was slightly higher at the station PV, with a difference of 0.1 $^{\circ }$C occurring in the period of maximum daily water temperature, which happens around 2 p.m. (Fig. 8). From the other depths, the differences between the LR and PV stations decreased even further (Fig. 9).

**Fig. 10:**
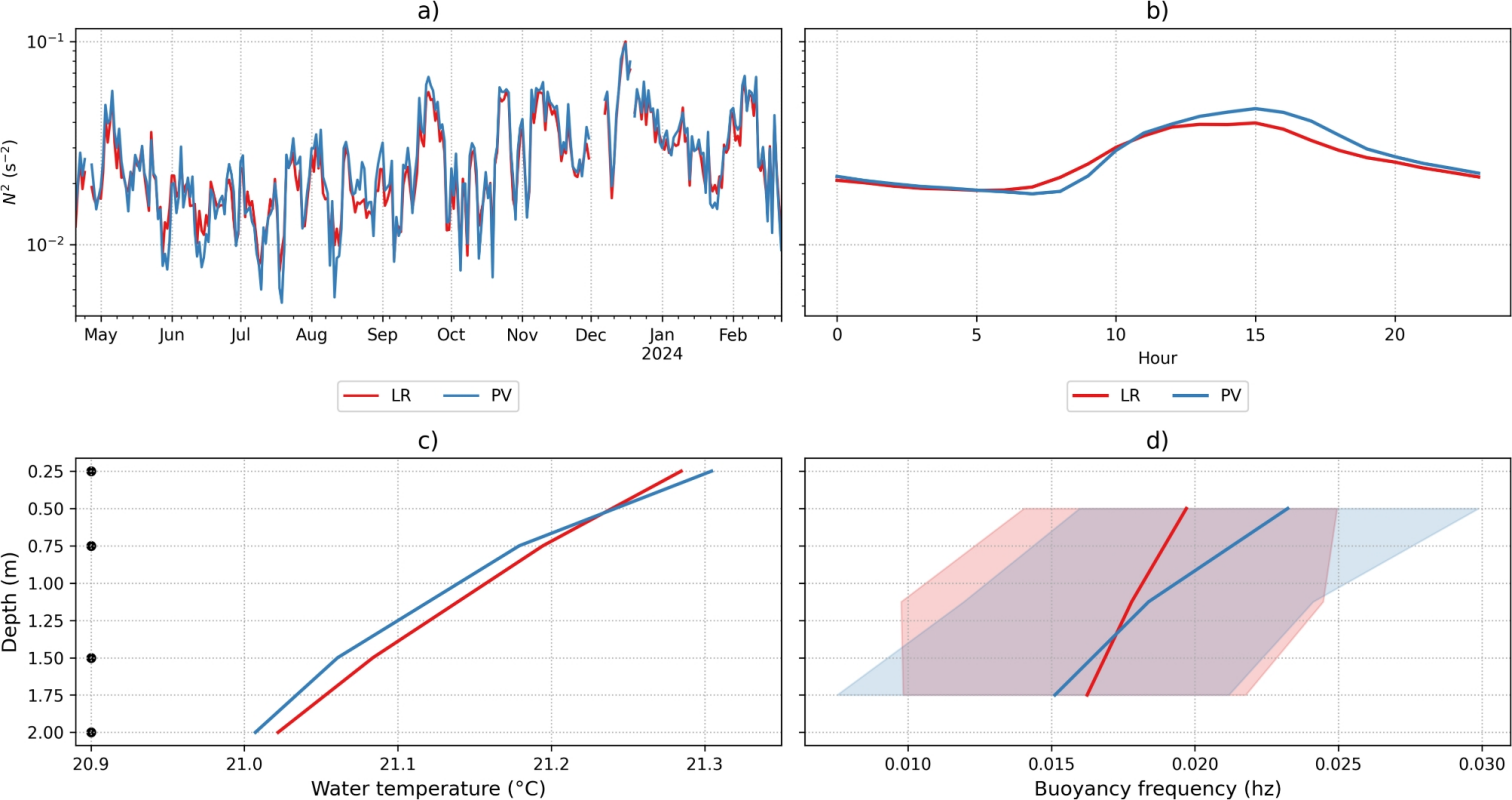
Analysis of vertical mixing. (**a**) Time series of buoyancy frequency ($N^2$). (**b**) Hourly average of buoyancy frequency throughout the day. (**c**) Mean temperature profile. (**d**) Mean buoyancy frequency profile.

From the higher temperature gradients observed in the temperature profiles, we obtained a greater buoyancy frequency ($N^2$) near the water surface, suggesting a more stable water column at the PV station, which indicates a resistance to mixing (Fig. 10a). Based on average hourly values, the buoyancy frequency reveals that the PV station is more stable than the LR station near the water surface, especially between 10 a.m. and 8 p.m. (Fig. 10b). At greater depths (> 0.50 $\textrm{m}$), the buoyancy frequency becomes similar, and the stability is comparable for both stations (Fig. 10c).

## Discussion

The most significant effect observed during the monitoring campaign was the decrease of the PAR, with the PV monitoring station showing on average 94.7% reduction compared to the reference station (Fig. 5b). This information can be relevant for setting up numerical models to estimate the effects of FPV systems with larger coverages, where still monitoring data is missing, or FPV systems in planning phase. Although this is a significant finding, its applicability to all FPV systems may be limited due to variations in FPV design, as indicated in previous studies^41,42^. Additionally, the measurements were conducted in the central part of the system, where radiation blocking is more pronounced compared to the edges of the photovoltaic panels. Irradiance can vary strongly depending on the location within the FPV^27^. Interestingly, the reduction in radiation did not directly result in a decrease in water temperature. The finding discussed below corroborates with previous studies, supporting the hypothesis that the substantial reduction in shortwave radiation caused by FPV coverage can be compensated by the increase in net longwave radiation, primarily attributed to the heat emissions from the panels^24^.

The temperature measurements provide valuable insights into the influence of FPV on lakes and reservoirs. While existing literature often focuses on binary outcomes, such as temperature increases or decreases below the FPV, it is essential to account for diurnal variations and the role of panel temperatures. Some studies report that FPV contributes to water temperature increases^10,24,43^, while others document decreases^1,22,23,26,27,44–46^. Additionally, no significant changes in water temperature were also reported^25^, emphasizing a lack of consensus across different studies. These discrepancies may be attributed to differences in monitoring or modeling methodologies, variations in FPV design, and the proportion of FPV coverage relative to the surface area of the water body.

Although no significant differences were observed between the PV and LR stations, the pilot FPV studied here, covering only 0.02% of the reservoir’s water surface, showed a tendency for the surface water at the PV station to warm, particularly during daily maximum temperatures. However, at greater depths, the temperature pattern reversed, with the LR station exhibiting higher temperatures as depth increased. This pattern aligns with the shading effect reported in previous studies^1,22,23,25–27,46^, which tends to intensify with depth.

The most significant temperature differences between FPV-influenced areas and those without reported a reduction of water temperature by 2.8 $^{\circ }$C^27^. Similarly, a decrease in water temperature was observed with more extensive FPV coverage, leading to a significant cooling effect^11^. They suggested that the FPV system could serve as a valuable tool for mitigating lake warming due to climate change^11^. However, these findings are based on one-dimensional modeling, where FPV effects were simulated by reducing forcing variables, primarily irradiance, neglecting all hydrodynamic effects (i.e. horizontal transport) that could be affected by FPV. While shading is a critical mechanism induced by FPV systems, other factors can also influence heat transfer. For instance, the elevated temperature of panels warming up significantly, depending on the FPV design, and the air between the FPV and the water surface increases heat flux from air to the water. As shown in Fig. 3, the time-averaged air temperature between the water surface and FPV (25 $^{\circ }$C) was higher than the time-averaged air temperature recorded at the weather station (20 $^{\circ }$C), highlighting the potential role of this mechanism for heat dynamics.

Following the slight apparent increase in water temperature induced by the FPV panels, the water column at the PV station (Fig. 10) exhibited a marginally higher buoyancy frequency compared to the LR station. This suggests a slightly increased resistance to mixing in the surface boundary layer. This phenomenon may result from a combination of factors: the warming of surface water due to heat flux from the warmer FPV panels, and a reduction in mechanical mixing caused by the FPV system, which partially obstructs wind fetch, thereby diminishing its contribution to vertical mixing.

Field measurements in Bomhofsplas Lake demonstrated that even for a large-scale FPV system with 30% surface coverage, temperature gradients showed minimal variations, with only a slight increase in temperature beneath the floating solar panels^43^. Similarly, continuous measurements in Lake Maiwald (Germany) revealed diurnal temperature differences between reference stations and those under the FPV; however, the surface boundary layer was not significantly altered, indicating that vertical mixing was not strongly affected by the FPV^27^. In contrast, temperature measurements in Tengeh and Poyan Reservoirs, with 30% FPV coverage, indicated a significant increase in temperature beneath the panels, leading to a notable increase in mixing resistance and a substantial reduction in the surface boundary layer^24^. These contrasting findings highlight the need for further research to identify the dominant mechanisms controlling vertical mixing in FPV-covered regions and to understand how FPV size and design influence these dynamics.

In this regard, numerical modeling approaches, particularly those based on three-dimensional simulations, can provide valuable insights into the impacts of various FPV coverage scenarios. These models allow for the exploration of how different coverage designs influence the hydrodynamic and temperature distributions of lakes and reservoirs, providing a deeper understanding of the mixing patterns induced by FPV. However, numerical results often exhibit significant divergence^11,24^, primarily due to a lack of consensus on how FPV should be represented in simulations. Many models overlook critical factors, such as the baroclinic forces that drive lake hydrodynamics, especially when results are based solely on vertical heat transfer, as is often the case with one-dimensional simulations^11,27^. Additionally, the heat flux from the warmer air beneath the panels to the surface water is frequently neglected, further limiting the accuracy of such models. Our results highlight the need to improve modeling approaches to better represent the key processes that influence the impact of FPV panels on lakes and reservoirs.

In the surface layer, the mean dissolved oxygen (DO) concentration did not differ significantly between the stations, with a mean difference of only 0.2 $\textrm{mg}\, L^{-1}$. However, the variability at the LR station was approximately 50 % higher than at the PV station, a trend that persisted throughout the entire analyzed period. Diurnal fluctuations were observed at both stations, but the variability was significantly greater at the LR station, with seasonal and daily components (Fig. 5a). Seasonal fluctuations revealed a wider range of DO concentrations during spring and autumn at the LR station. Although the diurnal component showed the highest peak during daylight hours at both stations, the variations in DO concentration were significantly greater at the LR station. This might be related to biofilm growth at the sensor, which was observed in November 2023. Nevertheless, the variability was observed even after in situ monitoring campaigns, where all the sensors were checked and cleaned (Fig. 6a). Previous studies on Lake Leimersheim have suggested that the reduced variability in dissolved oxygen concentration beneath FPV, also identified from field measurements, could be linked to mussels proliferating on the panel supports, acting as filtration organisms^47^. However, no such mussel communities were observed on the FPV supports in Passaúna reservoir. This suggests that the reduced fluctuations in DO under FPV panels in Passaúna reservoir could be attributed to diminished mixing, as wind shear beneath the panels is attenuated^27^. This attenuation creates resistance to mixing and reduces the dissipation of turbulent kinetic energy in the surface boundary layer driven by wind forcing. The diurnal component of the wind (Fig. 5b), which aligns with the DO fluctuations, supports this hypothesis. However, this study did not directly quantify wind speed within the FPV system, focusing instead on seasonal variations. Therefore, an in-depth analysis is needed to better understand the indirect mixing effects of wind under FPV systems. Moreover, measurements of gas exchange should be incorporated into future studies to explore the mechanisms behind FPV applications and their configurations more thoroughly. The floating structure in Passaúna reservoir has an area of 1326 $\textrm{m}^{2}$ which float configuration covers 51.5% of the FPV; in other words, almost half of the FPV does not fully block gas diffusion, that may explain the relatively small differences in DO concentration between the FPV and reference station.

Although fluctuations were more pronounced at the LR station, an interesting finding was the slightly higher DO values at LR from 9 a.m. to 8 p.m. (Fig. 6c), likely related to the daily cycle of primary production, which was more pronounced at the reference monitoring station. Passaúna reservoir is a subtropical reservoir classified as a discontinuous warm polymictic condition that could be associated with an efficient metabolism^48^. The mean chlorophyll concentration in different water depths within the euphotic zone was measured as 4.4 $\upmu \textrm{g}\, L^{-1}$, in addition, the reservoir presents a longitudinal gradient of chlorophyll superficial concentrations, that range from 5.0 $\upmu \textrm{g}\, L^{-1}$ to 30.2 $\upmu \textrm{g}\, L^{-1}$ in the lacustrine to riverine zones, respectively^49^. This gradient is related to high nutrient input from the watershed^50^. However, several mixing episodes observed over a year prevented the development of a consistent seasonal pattern in phytoplankton communities^49^. Both effects of photosynthesis on the differences of DO concentration, as well as the FPV effects on the reservoir ecosystem, should be further investigated in future research. The effects of FPV systems are neither strictly positive nor negative^25^ but are influenced by shading effects, which depend on the configuration of the floating structure (e.g., panel inclinations, effective coverage of supporting structures, wind-blocking efficiency, etc.).

## Conclusion

Floating photovoltaic systems can influence water temperature, even with the slight differences observed. Contrary to intuition, the PV station with the influence of the FPV showed a tendency to higher water temperatures at the surface, especially at the daily extremes. This trend towards higher surface temperatures could be related to less mixing of the water near the FPV. The lower mixing can lead to heat accumulation at the surface, resulting in higher temperatures. With increasing depth, the results suggest the potential of FPV to reduce water temperature in lakes or reservoirs. The water temperature proved to be challenging in terms of detecting differences caused by the FPV. However, even though the temperature differences comprise the uncertainty range of the sensors, some consistent trends were observed: at 0.25 $\textrm{m}$, a higher temperature trend at the PV station, being about 6% higher than at the LR station; the PSD also showed a higher energy, although small, about the water temperature at the PV point; and higher temperature due to less mixing, as indicated by the $N^2$, identifying greater stability in the water column at the PV point. At 0.50 $\textrm{m}$, the temperature pattern was similar to the 0.25 $\textrm{m}$ depth, with reduced effects. For greater depths (0.75 $\textrm{m}$, 1.50 $\textrm{m}$, and 2.00 $\textrm{m}$), the water temperature was consistently higher at the LR station with increasing depth. This effect suggests the shading effect of the FPV, which reduces the entry of solar radiation into the water column.

Due to the relatively small coverage of the FPV about the total superficial area of the reservoir (0.02%), the effects observed between the monitoring stations with and without FPV influence were small, except PAR. Photosynthetically active radiation at the LR station (without FPV influence) was significantly higher than at the PV station (below the FPV). The reduction in PAR at the PV station was 94.7%. This reduction could have important implications for photosynthesis and, consequently, for the phytoplankton community and the reservoir’s food web. These ecological implications should be detailed in further research.

The results of the increase or decrease in temperature in lakes or reservoirs due to FPV are still uncertain. Therefore, the data presented is fundamental to understanding the effects of FPV in a subtropical water supply reservoir and to help guide the future implementation of these systems. This study provides an understanding of the effects caused by FPV in aquatic environments, highlighting the small effect on temperature, but meaningful in terms of daily differences of DO and PAR, caused by the installation of the floating structure.

## Supplementary Information

Supplementary Information.

## Data Availability

The datasets used and/or analyzed during the current study available from the corresponding author on reasonable request.
