# Supplementary material for: High frequency monitoring for impact assessment of temperature, oxygen and radiation in floating photovoltaic system
Source: Sci Rep. 2025 Jun 5;15:19719. doi: 10.1038/s41598-025-96257-3 (PMC12137615; doi:10.1038/s41598-025-96257-3)
Supplement: Supplementary file 1 — Supplementary Information. [file 41598_2025_96257_MOESM1_ESM.pdf]

High frequency monitoring for impact assessment of temperature, oxygen and radiation in Floating Photovoltaic System

Matheus Kopp Prandini[1], **Rafael de Carvalho Bueno**[1], **Jucimara Andreza Rigotti**[2], **Tobias Bleninger**[1,*], **Michael Mannich**[1], **and Luis Henrique Novak**[3]

[1]Dept. of Environmental Engineering - DEA, Federal University of Paraná - UFPR. Caixa Postal 19011, 81531-990, Curitiba - PR, Brazil
[2]Postgraduate Program in Water Resources and Environmental Engineering, Federal University of Paraná - UFPR. Caixa Postal 19011, 81531-990, Curitiba - PR, Brazil
[3]Paraná State Water and Sanitation Company - SANEPAR. Rua Engenheiros Rebouças, 1376, 80215-900, Rebouças, Curitiba - PR, Brazil
[*]bleninger@ufpr.br

Supplementary material

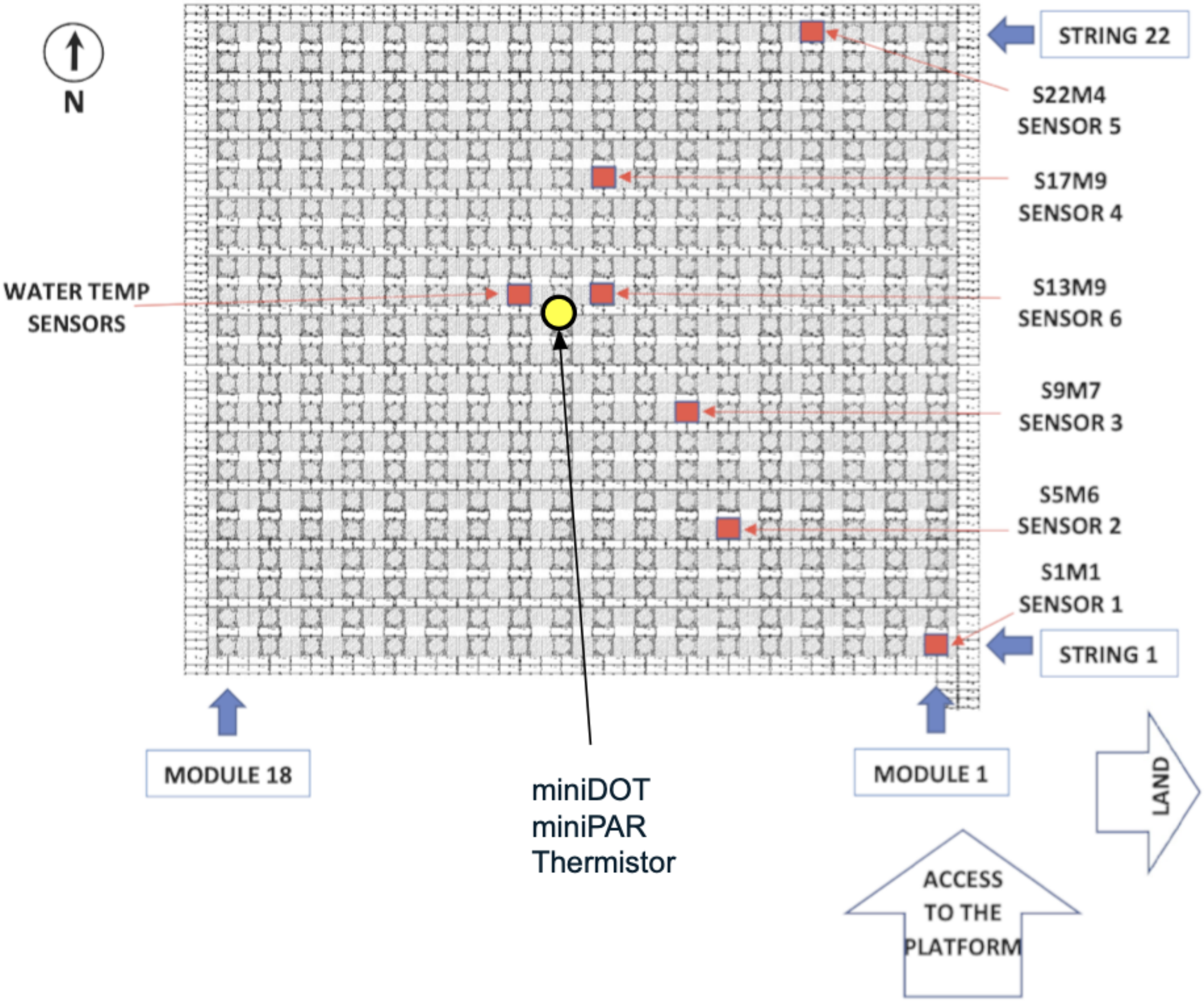

Figure S1. A detailed schematic diagram of the floating photovoltaic system, highlighting the specific locations of the temperature sensors on the photovoltaic modules (marked in red) and the central positioning of the miniDOT, miniPAR, and thermistor sensors (represented as a yellow point).

(a) Float of type 1. **(b)** Float of type 2.

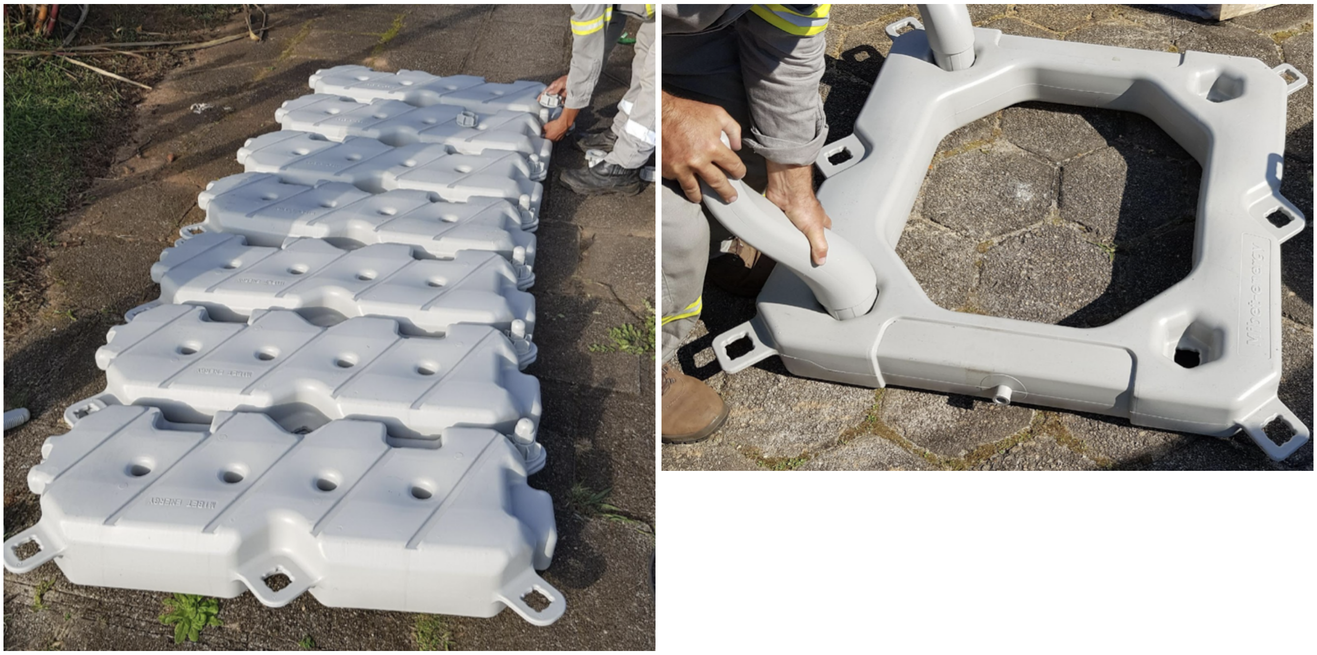

Figure S2. Types of floats

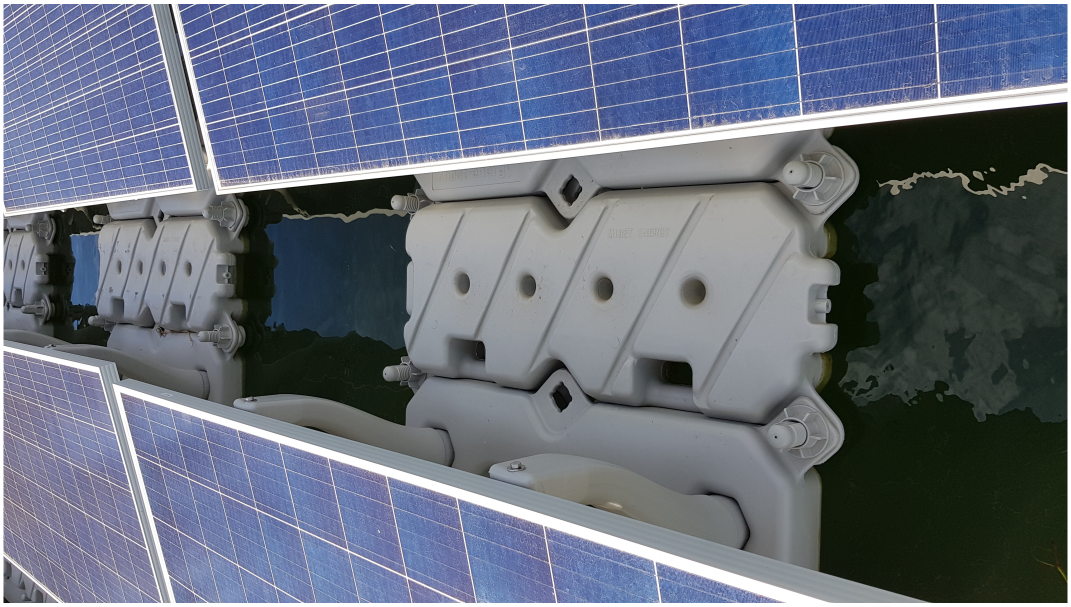

Figure S3. Configuration of floats in contact with water and free water surface.

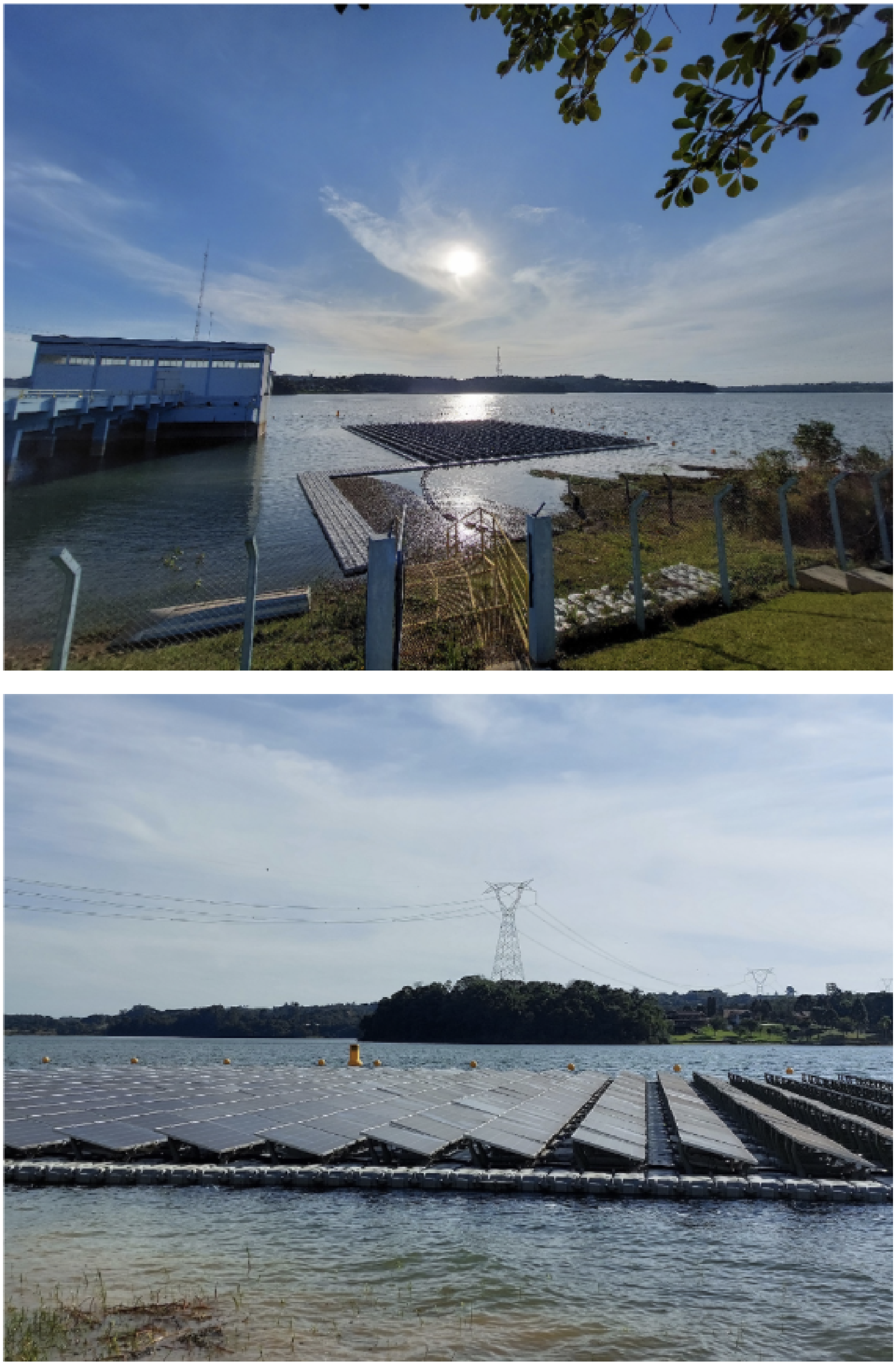

Figure S4. Comprehensive design framework of the floating photovoltaic system.

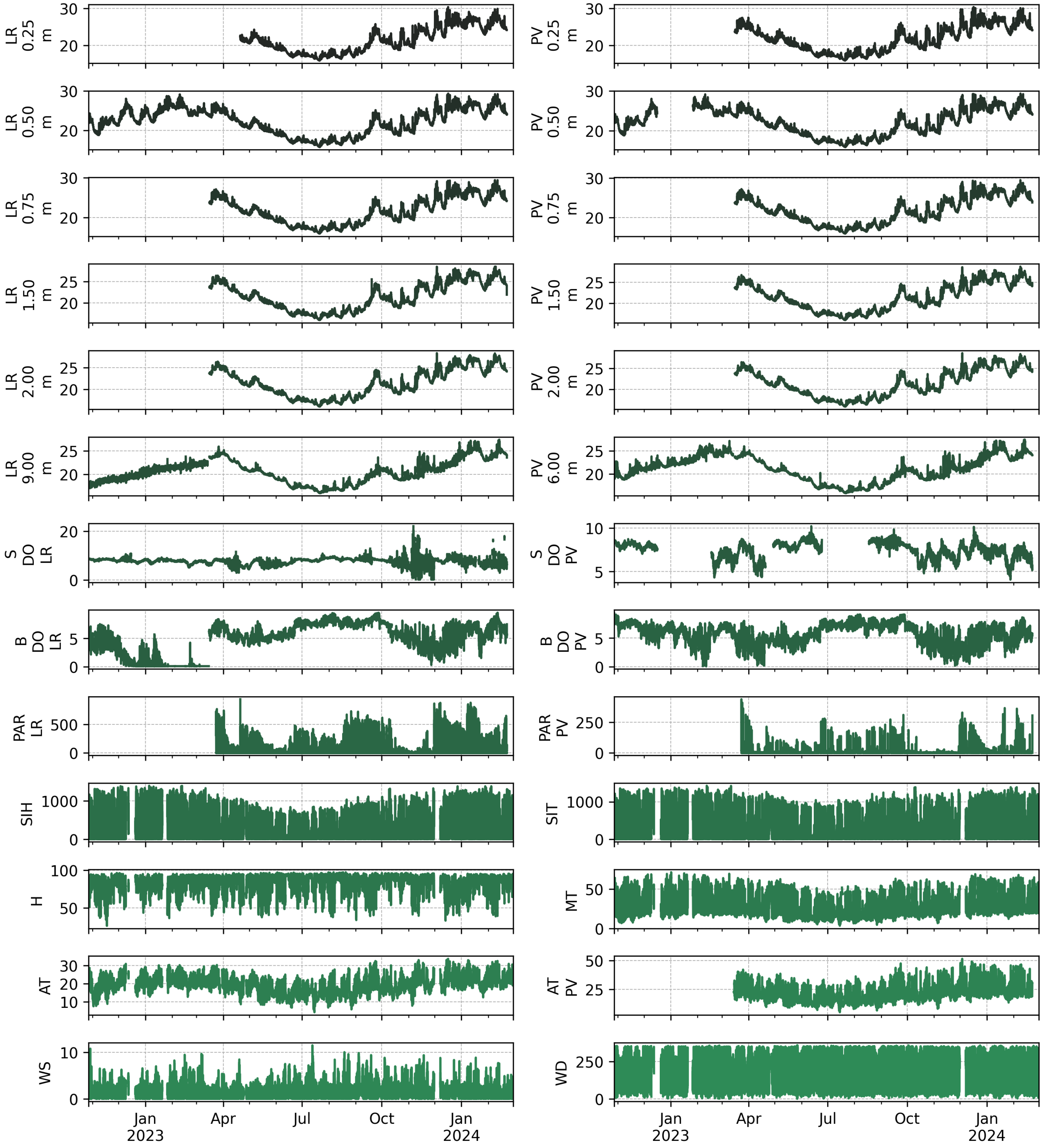

Figure S5. Time series data for the monitored environmental parameters.

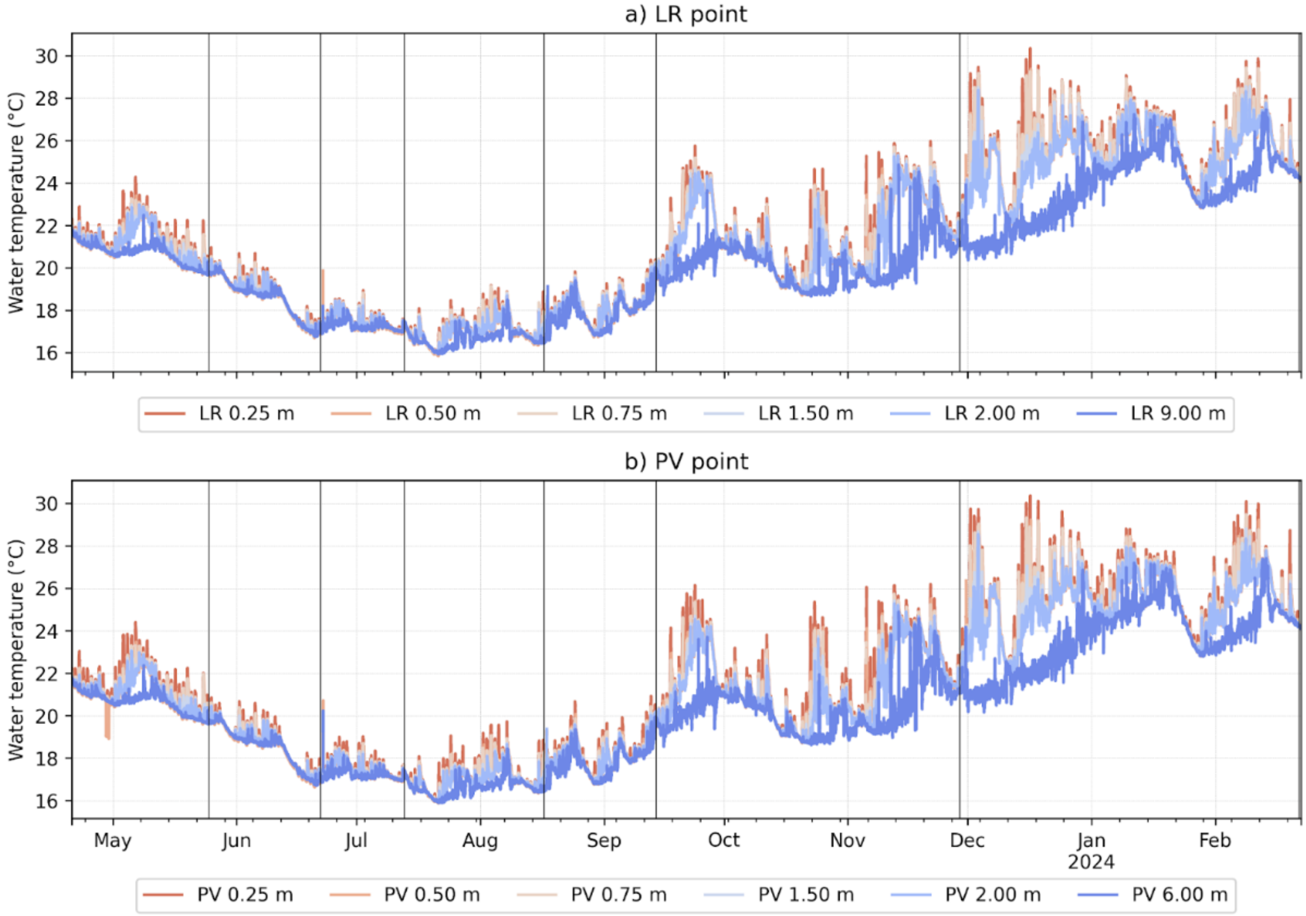

Figure S6. Time series of the water temperature profile for stations LR (a) and PV (b). Water temperature was measured at depths of 0.25 m, 0.50 m, 0.75 m, 1.50 m, and 2.00 m. Bottom water temperature was monitored at depths of 6.00 m and 9.00 m for stations PV and LR, respectively. Vertical dashed lines indicate periods of data collection and sensor maintenance campaigns.

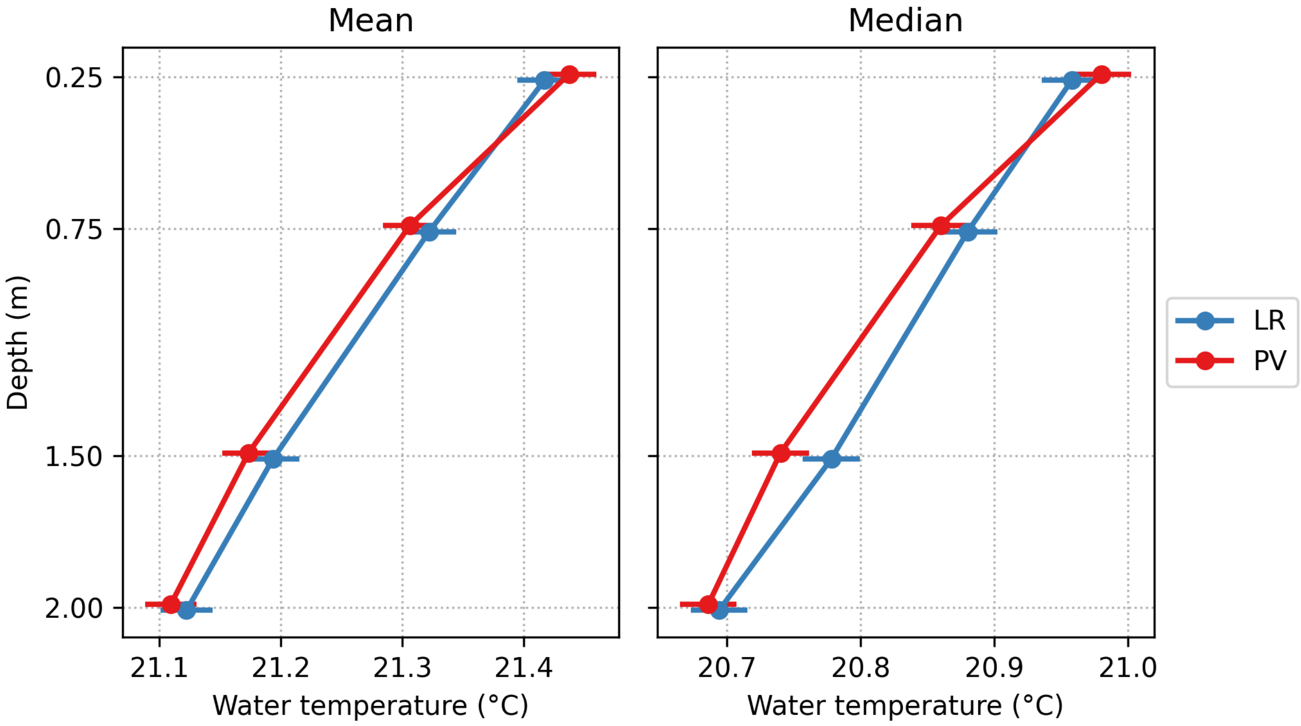

Figure S7. Mean and median values, accompanied by their respective 95 % confidence intervals, are calculated for the thermistor temperature profile, with a comparative assessment between the LR and PV points.
